# The Social Connectome – Moving Toward Complexity in the Study of Brain Networks and Their Interactions in Social Cognitive and Affective Neuroscience

**DOI:** 10.3389/fpsyt.2022.845492

**Published:** 2022-04-05

**Authors:** Lara Maliske, Philipp Kanske

**Affiliations:** Clinical Psychology and Behavioral Neuroscience, Faculty of Psychology, Technische Universität Dresden, Dresden, Germany

**Keywords:** social cognition, network neuroscience, connectome, network interaction, mental disorders

## Abstract

Over the past 150 years of neuroscientific research, the field has undergone a tremendous evolution. Starting out with lesion-based inference of brain function, functional neuroimaging, introduced in the late 1980s, and increasingly fine-grained and sophisticated methods and analyses now allow us to study the live neural correlates of complex behaviors in individuals and multiple agents simultaneously. Classically, brain-behavior coupling has been studied as an association of a specific area in the brain and a certain behavioral outcome. This has been a crucial first step in understanding brain organization. Social cognitive processes, as well as their neural correlates, have typically been regarded and studied as isolated functions and blobs of neural activation. However, as our understanding of the social brain as an inherently dynamic organ grows, research in the field of social neuroscience is slowly undergoing the necessary evolution from studying individual elements to how these elements interact and their embedding within the overall brain architecture. In this article, we review recent studies that investigate the neural representation of social cognition as interacting, complex, and flexible networks. We discuss studies that identify individual brain networks associated with social affect and cognition, interaction of these networks, and their relevance for disorders of social affect and cognition. This perspective on social cognitive neuroscience can highlight how a more fine-grained understanding of complex network (re-)configurations could improve our understanding of social cognitive deficits in mental disorders such as autism spectrum disorder and schizophrenia, thereby providing new impulses for methods of interventions.

## The Modular Social Brain

We live in a social world requiring constant behavioral adaptation to changing socio-environmental demands. Socio-affective and -cognitive functions have been distinguished as crucial for coping with these demands and appropriately updating behavior in social situations. Our understanding of how these processes are represented in the brain has evolved quite substantially. In the 19th century, researchers relied on lesion-based approaches to infer the coupling of brain areas and behavior [a prominent example is that of Phineas Gage, a railroad worker that received extensive damage to parts of the frontal lobe after a workplace accident, and showed pronounced personality changes, (1)]. The development of increasingly sophisticated methods of non-invasive functional neuroimaging starting from the end of the last century on allowed researchers to arrive at studying the online coupling of social processes and neural activation at certain areas of interest.

The past two decades have yielded somewhat of a consensus regarding the brain areas associated with socio-affective and -cognitive functions (2). These social processes and the associated neural activations have classically been investigated as isolated functions and related neural networks. The underlying assumption has been that specialized “social brain regions” can be identified, potentially leading to an atlas of specific brain regions associated with social processes. A strength of this first wave of social neuroscience viewing social affect and cognition as separate and modular is that it allowed researchers to identify “core elements” guiding behavior in social situations, such as empathy [the affective representation of another’s emotions, (3)] and Theory of Mind [ToM, the cognitive representation of others’ mental states, (4)].

**FIGURE 1:**
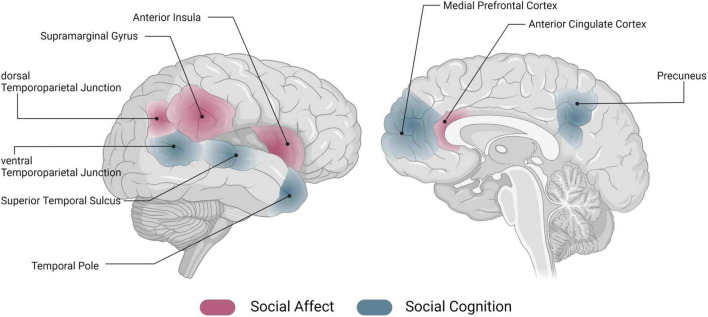
Networks of the social brain. Schematic overview of activation in regions that have previously been associated with social affect (red) and social cognition (blue) [for an overview, see e.g., (7–10)]. Image generated using biorender (www.biorender.com).

A large body of work has investigated the association of individual areas (such as the insula, temporoparietal junction, TPJ) with these socio-affective and -cognitive processes. While it has been crucial to build a base of understanding related to social affect and cognition as individual processes, it is becoming clearer that this does not seem to be the whole story. In naturalistic social interactions, we are confronted with a multitude of social and non-social information, which must be processed simultaneously to react appropriately [e.g., (5)]. Investigating pieces of social information processing in isolation, but also brain activation related to only one aspect of social information processing, appears to be too simplistic to understand actual social behavior. At the neural level, knowledge about the functional profiles of an individual area is the cornerstone on which to build on, however, understanding how an area is embedded within the overall brain architecture and how it is communicating with other areas of the brain can bring about another level of understanding [see (6) for an account of connectivity-based valence-specificity of the anterior insula]. Note that for reasons of simplicity, in the following we will refer to the neural representation of social affect and cognition, and the underlying neural networks [see e.g., (2, 7–10)], as “the social brain.” However, we want to stress that this is merely a simplification for illustrative purposes; we believe that the actual neural representation of social affective and cognitive processing requires an intricate pattern of interactions among components of the entire brain. Figure 1 gives an illustrative overview of regions previously associated with socio-affective and -cognitive processes.

A bit like moving from inspecting only a snippet of a painting to stepping back and observing it in its entirety, there is a chance to better understand and predict social behavior by considering the inherently interconnected, dynamic nature of information processing in the brain. Recent advances in the field of connectomics and network neuroscience – studying the brain in terms of a comprehensive map (11) – make methods that allow for a more holistic view of the brain accessible to a wider scientific community. In the following, we are employing ideas from the field of connectomics to describe interactions among modular brain networks associated with social affect and cognition [for a comprehensive introduction to the field of connectomics, see e.g., (11, 12)]. We want to highlight the added value of employing these ideas and methodology which allows to describe neural representation of social affect and cognition in terms of their network organization, especially for the field of (clinical) social cognitive neuroscience.

## The Connected Social Brain

The aim of the current review is to outline recent, promising avenues to describing the social brain at the network level, and how these networks interact in complex social situations.

### Network Organization of the Social Brain

Alcalá-López et al. (13) describe the social brain across a wide range of different (social and non-social) behavioral domains and experimental setups. The authors identified key social cognition hubs from the neuroimaging literature, and investigated neural networks associated with these hubs using meta-analytic connectivity modeling (MACM) and resting-state functional connectivity mapping. Furthermore, they performed hierarchical clustering and functional decoding of their identified networks to describe commonalities of the observed networks, as well as compare their results with a wide range of topics from neuroimaging research. We want to highlight two findings from their extensive analysis: (a) the authors identified a hierarchical organization of the social brain’s functional connectivity profiles into four dimensions (visual-sensory, limbic, intermediate-, and higher-level seeds). The authors observed considerable cross-network interactions for intermediate-level seeds, while the higher-level seeds displayed mostly connections within their respective network. Furthermore, the authors found (b) no one-to-one mapping for a seed region onto only one behavioral and experimental domain. In fact, the authors showed that activation in each seed region corresponded to a wide range of social and non-social topics, suggesting that the notion of specialized “social brain regions” is too simplistic [with possible exceptions like the fusiform face area, (14)].

In a similar vein, we performed a meta-analysis and hierarchical clustering analysis across empathy and ToM task groups (2). We observed a tripartite hierarchical organization of the networks associated with empathy and ToM tasks: neural activation of the task clusters typically associated with empathy and ToM corresponded well with previously described neural empathy and ToM networks [e.g., (9, 15)]. Interestingly, we also observed a third task cluster: this cluster was comprised of more complex social tasks including both affective and cognitive stimulus elements (e.g., inferring a character’s next actions based on their mental or emotional state). The neural activation pattern associated with this cluster showed activation in regions previously associated with empathy and ToM [see also (16)], networks previously described as independent [e.g., (17–19)].

While these studies only represent a small excerpt from the field of social affect and cognition, they summarize important new developments, showing how a more network-based perspective on social cognitive neuroscience can give new insights into the underlying neural architecture, but also the processes themselves [similar to other research areas, like intelligence, e.g., (20), or working memory, e.g., (21)].

### Interaction Between Networks of the Social Brain

Previous research has found substantial overlap between networks of social affect and cognition and selected canonical resting-state networks of the brain, which might enable closely related or compatible cognitive functions (22). A prime example for this is the overlap of the default mode network (DMN) with areas typically associated with ToM. The DMN is assumed to mediate self-generated cognition decoupled from the surrounding environment (23), which might be compatible with certain processes engaged during mental state reasoning. Recently, we systematically investigated the overlap of basic networks of the brain and social affect and cognition networks (24). We computed an overlap of canonical resting-state networks of the brain (25) with meta-analytically derived network maps associated with different social affect and cognition tasks. While ToM tasks primarily overlapped with the DMN [an interesting exception being the Reading the Mind in the Eyes task, see also (2)], overlap for complex social and empathy tasks was more heterogeneous: classical empathy tasks showed largest overlap with the ventral attention network (VAN), however there was also sizable overlap with other higher-level cognitive (e.g., frontoparietal network, FPN) and lower-sensory networks (e.g., visual network), pointing to increased cross-network interaction.

Having established that these seemingly independent neural networks do in fact interact, the next question might be how these networks interact. Studies of directed connectivity allow us to investigate how activity in one region causally influences activity in another region (26). A handful of studies investigated directed connectivity between regions of different canonical resting-state networks related to social cognition. Kanske et al. (18) found an inhibitory relationship from the insula (located in the FPN) onto the TPJ (located in the DMN) mediated by emotional content in a naturalistic social cognitive paradigm. Activity in the insula seemed to downregulate activity in the TPJ when participants viewed emotionally negative videos, which went along with impaired performance on an associated ToM-measure. The authors argue that this might be due to the emotional content of the video being more salient and requiring the most immediate response. Similarly, Regenbogen et al. (27) observed up-regulatory effects of a visual network onto a DMN region in the same experimental condition. In contrast, Schuwerk et al. (28) observed reciprocal down-regulation of DMN- and VAN-related regions for a false belief video task in conditions wherein a demonstrator and participant’s belief are incongruent. As a last example, social cues in an attentional re-orienting task (29) were associated with up-regulation of a VAN- onto a DMN-related region.

The interaction and reconfiguration of brain region interactions is rather complex and seems largely context-dependent [for a review, see (30)]. Rich, naturalistic social situations present us with a plethora of different cognitive and affective information, which must be processed simultaneously (31, 32). To react appropriately, certain information must be integrated while other information must be blocked out (33, 34). The notion of network integration, that is, the interaction of modular sub-components of different networks, has been associated with tasks that require more effortful and controlled processing (35), including more complex social tasks (36). Network integration might be a relevant mechanism, especially for complex and naturalistic social tasks, as it provides a means of integrating different mechanisms across unique behavioral domains (30).

### Malleability of the Connected Social Brain

Measures of network interaction cannot only be contextually reconfigured (30), but also altered by interventions or training. In one such study, Valk et al. (37) investigated reorganization in networks relevant to attention, socio-affective, and socio-cognitive processing after intensive 9-month meditation training, that did indeed improve behavioral measures of the respective functions (38). Using gradient-based approaches to measure network integration, Valk et al. (37) could show that training in different meditation practices went along with differential patterns of network reorganization. After socio-cognitive meditation training, the authors observed increased functional integration of regions in the DMN, FPN, and dorsal attention network (DAN). Furthermore, the organization of task-based neural networks associated with attention and social cognition became more similar to other networks within the overall connectome after socio-cognitive meditation training. Alterations in network organization after socio-affective meditation training resulted in increased network integration of areas of the VAN with the DMN, FPN, and DAN along the hierarchical organization of the first gradient. These changes in large-scale network organization could furthermore predict changes in behavioral ToM and compassion measures.

Taken together, these data can greatly enhance our understanding of (a) how the brain represents social affect and cognition, (b) the nature of social affect and cognition, and how they relate to one another, (c) the context-dependency of how the brain represents social affect and cognition, and (d) the flexibility, adaptability, and malleability of how the brain represents social affect and cognition. Just like social encounters in real-life interactions, the relationship between social affect and cognition, and their representation in the brain, is marked by a complex, interconnected pattern of excitatory and inhibitory connections.

## The Disconnected Social Brain

If we understand the organization of social processes in the brain in terms networks and argue that their interaction underlies successful social interactions, we should also be able to use this network-based perspective to enhance our understanding of failures and disorders of social cognition.

A growing body of research has investigated alterations of brain network organization in different neurological and mental disorders (39–41). Evidence suggests that brain structural and functional alterations associated with neurological and mental disorders are more likely to be located in densely interconnected regions of the brain (42) and at white matter pathways relevant for cross-network interaction and global communication (43). In the following, we want to pinpoint relevant studies discussing network-based alterations in subgroups of mental disorders that might shed light on disorder-specific and more general pathophysiological neural or behavioral dysfunctions.

Patients with bipolar disorder (BD) have been found to show altered socio-cognitive and emotional processing and perception (44), which has furthermore been associated with altered functioning in a central-limbic network and decreased activity in dorsal brain areas (45, 46). Recently, Roberts et al. (47) found decreased structural connectivity in networks centered on the inferior frontal gyrus and left insular cortex in youths at high risk for developing BD, as well as increased connectivity in the limbic network. These regions have been shown to be implicated in altered neural functioning in individuals with BD (48) and are also core regions associated with socio-affective and -cognitive functioning (2). A recent study associated altered functional connectivity with socio-cognitive task performance in participants with BD and schizophrenia (49). Here, the authors compared network connectivity between patients with BD, schizophrenia, and healthy controls, and related network connectivity to measures of social affect and cognition. Altered network connectivity was observed in networks related to visual processing in BD and schizophrenia, which was associated with differential performance in socio-cognitive tasks in both patient groups. The authors argue that compensatory mechanisms might cushion behavioral deficits in BD, while this was not the case for participants with schizophrenia, elegantly demonstrating how measures of network organization might be a transdiagnostic marker of socio-cognitive deficits in mental disorders.

It is generally agreed upon that brain network organization is altered in individuals with autism spectrum disorder (ASD), however, the nature of these alterations remains a topic of ongoing debate. It seems that neural patterns of connectivity in individuals with ASD are a complex phenotype, with studies reporting both hyper- and hypoconnectivity [e.g., (50, 51); for a developmental account, see (52)], as well as reduced functional integration and segregation in networks related to social information processing (53). A recent study comparing functional connectivity in a complex social task across ASD, attention-deficit/hyperactivity disorder, and a comorbid group found distinctly altered connectivity profiles related to socio-cognitive processing (54). More specifically, while the three groups did not differ in terms of task performance, they did show decreased connectivity in a key region of what the authors term the social cognitive network (centered on the right temporoparietal cortex). Participants with ASD showed decreased connectivity between nodes of this network, which the authors attribute to atypical informational transfer during social cognition.

These studies highlight how a network-based perspective might explain previously heterogeneous findings, or might shed light onto underlying mechanisms associated with altered neural processing and overall social cognitive dysfunctions. Especially moving toward fully socially interactive experimental paradigms to better understand real-life social deficits will necessitate more complex analyses of the related brain activity (55). Additionally, network-based characteristics might serve as an additional transdiagnostic marker of mental disorders of social cognition, in line with the growing interest in dimensional approaches to mental disorders [like the RDoC framework, (56)], or might offer up new explanatory models for mental disorders (57).

## The Future of the Connected Social Brain

Our understanding of the processes underlying social affect and cognition, as well as how they are represented in the brain has undergone a tremendous evolution. From a modular, isolated understanding of these processes, the field has now arrived at a more interconnected, complex view. Among others, developments in methodology are making more advanced analyses and representations of social brain activity accessible to the scientific community. Overall, the field is moving from the search of an individual “social seed” in the brain (areas specifically dedicated to orchestrating one specific function) toward a more large-scale investigation of how the social brain is organized.

In this review, we highlighted individual studies that showcase how this move toward a network-based investigation of the social brain might reshape our understanding of social affect and cognition in terms of overall network organization (2, 13), network configuration and interaction (18, 27), flexible network reconfiguration (37), and disorders of the social brain (49, 54). This approach offers promising avenues for the field of (clinical) social neuroscience, which will allow us to gain a more holistic understanding of how the brain represents social processing, and social processing in itself. Analogous to the move toward a second-person neuroscience (58), moving toward a network-based perspective of the social brain might help sharpen our understanding of different areas of the brain as interacting, interconnected networks.

Beyond basic research, a move toward a network-based understanding of the social brain could open up crucial avenues especially in the context of clinical research, similar to how connectome-based decoding is now used in, for example, neurological or psychiatric research. To illustrate, in neurological research, information about connectome-level organization of the brain was successful in diagnosing disorders or predicting long-term outcome (59, 60). But also in the context of mental health, information about connectome organization has been shown to aid diagnosis of disorders (61), classify patient subgroups (62), and predict symptom severity (63). Correspondingly, information about the connectome-level representation of social affect and cognition in the brain might help to predict alterations in interpersonal behavior and social cognitive functioning associated with a wide range of mental disorders. Similar to the approach of precision medicine, adopting a perspective of “precision connectomics” could support clinical work substantially (57).

While the classically held view of an isolated and modular social brain paved the way for our currently held understanding of social affect and cognition, we believe that the field is ready to move toward a more holistic account of the social brain – in terms of both, how we probe social affective and cognitive processing (the employed task paradigms) and how we map their neural representation. Adopting a network-based perspective on social affect and cognition cannot only enhance our understanding of the social brain itself, but also of the underlying processes, their relationship with each other, and possible alterations in them.

## Author Contributions

LM and PK conceived the manuscript and contributed to writing and editing. Both authors contributed to the article and approved the submitted version.

## Conflict of Interest

The authors declare that the research was conducted in the absence of any commercial or financial relationships that could be construed as a potential conflict of interest.

## Publisher’s Note

All claims expressed in this article are solely those of the authors and do not necessarily represent those of their affiliated organizations, or those of the publisher, the editors and the reviewers. Any product that may be evaluated in this article, or claim that may be made by its manufacturer, is not guaranteed or endorsed by the publisher.

## References

[1] Damasio H Grabowski T Frank R Galaburda AM Damasio AR. The return of Phineas Gage: clues about the brain from the skull of a famous patient. *Science.* (1994) 264:1102–5. 10.1126/science.8178168 8178168

[2] Schurz M Radua J Tholen MG Maliske L Margulies DS Mars RB Toward a hierarchical model of social cognition: a neuroimaging meta-analysis and integrative review of empathy and theory of mind. *Psychol Bull.* (2021) 147:293–327. 10.1037/bul0000303 33151703

[3] Gallese V. The roots of empathy: the shared manifold hypothesis and the neural basis of intersubjectivity. *Psychopathology.* (2003) 36:171–80. 10.1159/000072786 14504450

[4] Kanske P. The social mind: disentangling affective and cognitive routes to understanding others. *Interdiscip Sci Rev.* (2018) 43:115–24. 10.1080/03080188.2018.1453243

[5] van Ackeren MJ Smaragdi A Rueschemeyer S-A. Neuronal interactions between mentalising and action systems during indirect request processing. *Soc Cogn Affect Neurosci.* (2016) 11:1402–10. 10.1093/scan/nsw062 27131039 PMC5015811

[6] Lamm C Singer T. The role of anterior insular cortex in social emotions. *Brain Struct Funct.* (2010) 214:579–91. 10.1007/s00429-010-0251-3 20428887

[7] Schurz M Radua J Aichhorn M Richlan F Perner J. Fractionating theory of mind: a meta-analysis of functional brain imaging studies. *Neurosci Biobehav Rev.* (2014) 42:9–34. 10.1016/j.neubiorev.2014.01.009 24486722

[8] Fan Y Duncan NW de Greck M Northoff G. Is there a core neural network in empathy? An fMRI based quantitative meta-analysis. *Neurosci Biobehav Rev.* (2011) 35:903–11. 10.1016/j.neubiorev.2010.10.009 20974173

[9] Bzdok D Schilbach L Vogeley K Schneider K Laird AR Langner R Parsing the neural correlates of moral cognition: ALE meta-analysis on morality, theory of mind, and empathy. *Brain Struct Funct.* (2012) 217:783–96. 10.1007/s00429-012-0380-y 22270812 PMC3445793

[10] Preckel K Kanske P Singer T. On the interaction of social affect and cognition: empathy, compassion and theory of mind. *Curr Opin Behav Sci.* (2018) 19:1–6. 10.1016/j.cobeha.2017.07.010

[11] Bassett DS Sporns O. Network neuroscience. *Nat Neurosci.* (2017) 20:353–64. 28230844 10.1038/nn.4502 PMC5485642

[12] Sporns O Tononi G Kötter R. The human connectome: a structural description of the human brain. *PLoS Comput Biol.* (2005) 1:e42. 10.1371/journal.pcbi.0010042 16201007 PMC1239902

[13] Alcalá-López D Smallwood J Jefferies E Van Overwalle F Vogeley K Mars RB Computing the social brain connectome across systems and states. *Cereb Cortex.* (2018) 28:2207–32. 10.1093/cercor/bhx121 28521007

[14] Weiner KS Grill-Spector K. The improbable simplicity of the fusiform face area. *Trends Cogn Sci.* (2012) 16:251–4. 10.1016/j.tics.2012.03.003 22481071

[15] Molenberghs P Johnson H Henry JD Mattingley JB. Understanding the minds of others: a neuroimaging meta-analysis. *Neurosci Biobehav Rev.* (2016) 65:276–91. 10.1016/j.neubiorev.2016.03.020 27073047

[16] Maliske L Schurz M Kanske P. Interactions within the social brain: co-activation and connectivity among networks enabling empathy and theory of mind. *PsyArXiv* [Preprint]. (2021). 10.31234/osf.io/52z4f 36764638

[17] Dziobek I Fleck S Kalbe E Rogers K Hassenstab J Brand M Introducing MASC: a movie for the assessment of socia cognition. *J Autism Dev Disord.* (2006) 36:623–36. 10.1007/s10803-006-0107-0 16755332

[18] Kanske P Böckler A Trautwein F-M Parianen Lesemann FH Singer T. Are strong empathizers better mentalizers? Evidence for independence and interaction between the routes of social cognition. *Soc Cogn Affect Neurosci.* (2016) 11:1383–92. 10.1093/scan/nsw052 27129794 PMC5015801

[19] Shamay-Tsoory SG Aharon-Peretz J Perry D. Two systems for empathy: a double dissociation between emotional and cognitive empathy in inferior frontal gyrus versus ventromedial prefrontal lesions. *Brain.* (2009) 132:617–27. 10.1093/brain/awn279 18971202

[20] Barbey AK. Network neuroscience theory of human intelligence. *Trends Cogn Sci.* (2018) 22:8–20. 10.1016/j.tics.2017.10.001 29167088

[21] Cohen JR D’Esposito M. The segregation and integration of distinct brain networks and their relationship to cognition. *J Neurosci Off J Soc Neurosci.* (2016) 36:12083–94. 10.1523/JNEUROSCI.2965-15.2016 27903719 PMC5148214

[22] Kanske P Böckler A Trautwein F-M Singer T. Dissecting the social brain: introducing the EmpaToM to reveal distinct neural networks and brain-behavior relations for empathy and theory of mind. *Neuroimage.* (2015) 122:6–19. 10.1016/j.neuroimage.2015.07.082 26254589

[23] Andrews-Hanna JR Saxe R Yarkoni T. Contributions of episodic retrieval and mentalizing to autobiographical thought: evidence from functional neuroimaging, resting-state connectivity, and fMRI meta-analyses. *Neuroimage.* (2014) 91:324–35. 10.1016/j.neuroimage.2014.01.032 24486981 PMC4001766

[24] Schurz M Maliske L Kanske P. Cross-network interactions in social cognition: a review of findings on task related brain activation and connectivity. *Cortex J Devoted Study Nerv Syst Behav.* (2020) 130:142–57. 10.1016/j.cortex.2020.05.006 32653744

[25] Yeo BTT Krienen FM Sepulcre J Sabuncu MR Lashkari D Hollinshead M The organization of the human cerebral cortex estimated by intrinsic functional connectivity. *J Neurophysiol.* (2011) 106:1125–65. 10.1152/jn.00338.2011 21653723 PMC3174820

[26] Friston KJ Harrison L Penny W. Dynamic causal modelling. *Neuroimage.* (2003) 19:1273–302. 10.1016/s1053-8119(03)00202-7 12948688

[27] Regenbogen C Habel U Kellermann T. Connecting multimodality in human communication. *Front Hum Neurosci.* (2013) 7:754. 10.3389/fnhum.2013.00754 24265613 PMC3820976

[28] Schuwerk T Döhnel K Sodian B Keck IR Rupprecht R Sommer M. Functional activity and effective connectivity of the posterior medial prefrontal cortex during processing of incongruent mental states. *Hum Brain Mapp.* (2014) 35:2950–65. 10.1002/hbm.22377 24115202 PMC6869201

[29] Schuwerk T Schurz M Müller F Rupprecht R Sommer M. The rTPJ’s overarching cognitive function in networks for attention and theory of mind. *Soc Cogn Affect Neurosci.* (2017) 12:157–68. 10.1093/scan/nsw163 27798260 PMC5390694

[30] Shine JM Poldrack RA. Principles of dynamic network reconfiguration across diverse brain states. *Neuroimage.* (2018) 180(Pt B):396–405. 10.1016/j.neuroimage.2017.08.010 28782684

[31] Schilbach L Eickhoff SB Mojzisch A Vogeley K. What’s in a smile? Neural correlates of facial embodiment during social interaction. *Soc Neurosci.* (2008) 3:37–50. 10.1080/17470910701563228 18633845

[32] Zaki J Weber J Bolger N Ochsner K. The neural bases of empathic accuracy. *Proc Natl Acad Sci U.S.A.* (2009) 106:11382–7. 10.1073/pnas.0902666106 19549849 PMC2708723

[33] Anticevic A Cole MW Murray JD Corlett PR Wang X-J Krystal JH. The role of default network deactivation in cognition and disease. *Trends Cogn Sci.* (2012) 16:584–92. 10.1016/j.tics.2012.10.008 23142417 PMC3501603

[34] Goulden N Khusnulina A Davis NJ Bracewell RM Bokde AL McNulty JP The salience network is responsible for switching between the default mode network and the central executive network: replication from DCM. *Neuroimage.* (2014) 99:180–90. 10.1016/j.neuroimage.2014.05.052 24862074

[35] Fornito A Harrison BJ Zalesky A Simons JS. Competitive and cooperative dynamics of large-scale brain functional networks supporting recollection. *Proc Natl Acad Sci U.S.A.* (2012) 109:12788–93. 10.1073/pnas.1204185109 22807481 PMC3412011

[36] Shine JM Bissett PG Bell PT Koyejo O Balsters JH Gorgolewski KJ The dynamics of functional brain networks: integrated network states during cognitive task performance. *Neuron.* (2016) 92:544–54. 10.1016/j.neuron.2016.09.018 27693256 PMC5073034

[37] Valk SL Kanske P Park B Hong SJ Böckler-Raettig A Trautwein F-M Changing the social brain: plasticity along macro-scale axes of functional connectivity following social mental training. *biorxiv* [Preprint]. (2021). 10.1101/2020.11.11.377895

[38] Trautwein F-M Kanske P Böckler A Singer T. Differential benefits of mental training types for attention, compassion, and theory of mind. *Cognition.* (2020) 194:104039. 10.1016/j.cognition.2019.104039 31450018 PMC6891878

[39] Alexander-Bloch A Gogtay N Meunier D Birn R Clasen L Lalonde F Disrupted modularity and local connectivity of brain functional networks in childhood-onset schizophrenia. *Front Syst Neurosci.* (2010) 4:147. 10.3389/fnsys.2010.00147 21031030 PMC2965020

[40] Lee K Khoo HM Lina J-M Dubeau F Gotman J Grova C. Disruption, emergence and lateralization of brain network hubs in mesial temporal lobe epilepsy. *Neuroimage Clin.* (2018) 20:71–84. 10.1016/j.nicl.2018.06.029 30094158 PMC6070692

[41] Wise T Marwood L Perkins AM Herane-Vives A Joules R Lythgoe DJ Instability of default mode network connectivity in major depression: a two-sample confirmation study. *Transl Psychiatry.* (2017) 7:e1105–1105. 10.1038/tp.2017.40 28440813 PMC5416685

[42] Crossley NA Mechelli A Scott J Carletti F Fox PT McGuire P The hubs of the human connectome are generally implicated in the anatomy of brain disorders. *Brain.* (2014) 137:2382–95. 10.1093/brain/awu132 25057133 PMC4107735

[43] de Lange SC Scholtens LH van den Berg LH Boks MP Bozzali M Cahn W Shared vulnerability for connectome alterations across psychiatric and neurological brain disorders. *Nat Hum Behav.* (2019) 3:988–98. 10.1038/s41562-019-0659-6 31384023

[44] Bora E Bartholomeusz C Pantelis C. Meta-analysis of theory of mind (ToM) impairment in bipolar disorder. *Psychol Med.* (2016) 46:253–64. 10.1017/S0033291715001993 26456502

[45] Kurtz M Mohring P Förster K Bauer M Kanske P. Deficits in explicit emotion regulation in bipolar disorder: a systematic review. *Int J Bipolar Disord.* (2021) 9:15. 10.1186/s40345-021-00221-9 33937951 PMC8089068

[46] Wessa M Linke J. Emotional processing in bipolar disorder: behavioural and neuroimaging findings. *Int Rev Psychiatry.* (2009) 21:357–67. 10.1080/09540260902962156 20374149

[47] Roberts G Perry A Lord A Frankland A Leung V Holmes-Preston E Structural dysconnectivity of key cognitive and emotional hubs in young people at high genetic risk for bipolar disorder. *Mol Psychiatry.* (2018) 23:413–21. 10.1038/mp.2016.216 27994220 PMC5794888

[48] Wessa M Kanske P Linke J. Bipolar disorder: a neural network perspective on a disorder of emotion and motivation. *Restor Neurol Neurosci.* (2014) 32:51–62. 10.3233/RNN-139007 23603441

[49] Jimenez AM Riedel P Lee J Reavis EA Green MF. Linking resting-state networks and social cognition in schizophrenia and bipolar disorder. *Hum Brain Mapp.* (2019) 40:4703–15. 10.1002/hbm.24731 31322784 PMC6865798

[50] Haghighat H Mirzarezaee M Araabi BN Khadem A. Functional networks abnormalities in autism spectrum disorder: age-related hypo and hyper connectivity. *Brain Topogr.* (2021) 34:306–22. 10.1007/s10548-021-00831-7 33905003

[51] Seghatol-Eslami VC Maximo JO Ammons CJ Libero LE Kana RK. Hyperconnectivity of social brain networks in autism during action-intention judgment. *Neuropsychologia.* (2020) 137:107303. 10.1016/j.neuropsychologia.2019.107303 31837376

[52] Uddin L Supekar K Menon V. Reconceptualizing functional brain connectivity in autism from a developmental perspective. *Front Hum Neurosci.* (2013) 7:458. 10.3389/fnhum.2013.00458 23966925 PMC3735986

[53] Rudie JD Shehzad Z Hernandez LM Colich NL Bookheimer SY Iacoboni M Reduced functional integration and segregation of distributed neural systems underlying social and emotional information processing in autism spectrum disorders. *Cereb Cortex.* (2012) 22:1025–37. 10.1093/cercor/bhr171 21784971 PMC3328339

[54] Ilzarbe D Lukito S Moessnang C O’Daly OG Lythgoe DJ Murphy CM Neural correlates of theory of mind in autism spectrum disorder, attention-deficit/hyperactivity disorder, and the comorbid condition. *Front Psychiatry.* (2020) 11:544482. 10.3389/fpsyt.2020.544482 33240117 PMC7677232

[55] Lehmann K Maliske L Böckler A Kanske P. Social impairments in mental disorders: Recent developments in studying the mechanisms of interactive behavior. *Clin Psychol Eur.* (2019) 1:1–15.

[56] Cuthbert BN. The RDoC framework: facilitating transition from ICD/DSM to dimensional approaches that integrate neuroscience and psychopathology. *World Psychiatry.* (2014) 13:28–35. 10.1002/wps.20087 24497240 PMC3918011

[57] van den Heuvel MP Sporns O. A cross-disorder connectome landscape of brain dysconnectivity. *Nat Rev Neurosci.* (2019) 20:435–46. 10.1038/s41583-019-0177-6 31127193 PMC8864539

[58] Schilbach L Timmermans B Reddy V Costall A Bente G Schlicht T Toward a second-person neuroscience. *Behav Brain Sci.* (2013) 36:393–414. 10.1017/s0140525x12000660 23883742

[59] Mirzaei G Adeli A Adeli H. Imaging and machine learning techniques for diagnosis of Alzheimer’s disease. *Rev Neurosci.* (2016) 27:857–70. 10.1515/revneuro-2016-0029 27518905

[60] van der Burgh HK Schmidt R Westeneng H-J de Reus MA van den Berg LH van den Heuvel MP. Deep learning predictions of survival based on MRI in amyotrophic lateral sclerosis. *Neuroimage Clin.* (2017) 13:361–9. 10.1016/j.nicl.2016.10.008 28070484 PMC5219634

[61] Brown M Sidhu G Greiner R Asgarian N Bastani M Silverstone P ADHD-200 global competition: diagnosing ADHD using personal characteristic data can outperform resting state fMRI measurements. *Front Syst Neurosci.* (2012) 6:69. 10.3389/fnsys.2012.00069 23060754 PMC3460316

[62] Schnack HG Nieuwenhuis M van Haren NEM Abramovic L Scheewe TW Brouwer RM Can structural MRI aid in clinical classification? A machine learning study in two independent samples of patients with schizophrenia, bipolar disorder and healthy subjects. *Neuroimage.* (2014) 84:299–306. 10.1016/j.neuroimage.2013.08.053 24004694

[63] Ramasubbu R Brown MRG Cortese F Gaxiola I Goodyear B Greenshaw AJ Accuracy of automated classification of major depressive disorder as a function of symptom severity. *Neuroimage Clin.* (2016) 12:320–31. 10.1016/j.nicl.2016.07.012 27551669 PMC4983635

